# Utilization and Operational Performance of the 988 Suicide & Crisis Lifeline in Nevada: A Retrospective Descriptive Analysis

**DOI:** 10.7759/cureus.107467

**Published:** 2026-04-21

**Authors:** Oscar A Toro Ruilowa, Deanna Chea, Justin B Atkins, Lisa Durette

**Affiliations:** 1 Medical School, University of Nevada, Las Vegas School of Medicine, Las Vegas, USA; 2 Department of Psychiatry, University of Nevada, Las Vegas School of Medicine, Las Vegas, USA

**Keywords:** 988 suicide & crisis lifeline, behavioral health crisis response system, behavioral health crisis systems, crisis hotlines, crisis intervention, health services research, mental health infrastructure, nevada public health, state health policy, suicide prevention

## Abstract

Background

The 988 Suicide & Crisis Lifeline, implemented nationally in 2022, was designed to improve access to immediate crisis counseling and suicide prevention services. Describing state-level operational metrics provides insight into how the national behavioral health crisis response system functions across diverse geographic and healthcare environments. Nevada represents a relevant setting due to its elevated suicide rates and large rural population.

Methodology

This retrospective descriptive study analyzed publicly available operational performance reports from the 988 Suicide & Crisis Lifeline network for Nevada from January through November 2025. Monthly operational metrics were analyzed descriptively, including call volume, in-state answer rates, call abandonment rates, average speed to answer, and average talk time. Trends were assessed descriptively to identify temporal patterns in system utilization and operational performance. National survey data were reviewed to provide a broader context regarding public awareness and perceived effectiveness of the 988 system, without direct comparison to Nevada-specific operational metrics due to differences in regional infrastructure and resources.

Results

Nevada’s 988 crisis hotline demonstrated sustained utilization during the study period, with more than 3,200 calls routed in-state each month and a total of 42,268 calls overall. Call volume showed moderate seasonal variation, peaking during the summer months. In-state answer rates ranged from 73.19% to 88.67%, while call abandonment rates remained relatively stable between 10.53% and 12.06%. Average speed to answer remained within several tens of seconds, and average talk time increased during peak utilization months. National survey findings indicate high public awareness of the 988 Lifeline, though fewer individuals report understanding how the service should be used during a behavioral health crisis.

Conclusions

Nevada’s 988 crisis hotline demonstrates sustained utilization and consistent operational trends, supporting its role as a key component of the state’s behavioral health crisis response infrastructure. Seasonal variation in demand and gaps in public understanding highlight opportunities to strengthen crisis center workforce capacity, improve public education regarding crisis resources, and enhance coordination across the behavioral health crisis care continuum.

## Introduction

Suicide remains a major public health challenge in the United States. In 2023, more than 49,000 suicide deaths were recorded nationally, representing the highest number ever reported and underscoring suicide as a leading cause of death, particularly among adolescents and young adults [1]. Suicide is also a major contributor to overall mortality in the United States, reflecting its significant public health burden [2]. Suicide prevention, therefore, remains a critical priority for public health systems, healthcare providers, and community organizations. In response to this growing burden, crisis intervention services have become an increasingly important component of suicide prevention efforts, providing immediate support for individuals in acute distress. The implementation of the 988 Suicide & Crisis Lifeline represents a national effort to expand access to these services; however, state-level data describing system utilization and operational trends remain limited.

Crisis intervention services have long served as an essential component of suicide prevention strategies by providing immediate access to trained counselors during periods of acute emotional distress. Suicide prevention hotlines are designed to deliver real-time emotional support, conduct risk assessment, and facilitate connection to appropriate mental health resources for individuals experiencing suicidal ideation or other behavioral health crises.

A growing body of research demonstrates that interactions with trained crisis counselors can significantly reduce emotional distress and suicidal ideation during crisis calls. Observational studies evaluating suicide prevention hotline interactions have shown that callers frequently report improvements in mood, reduced suicidal urgency, and increased feelings of hope during the course of the call [3,4]. Systematic reviews of crisis hotline interventions similarly conclude that these services play an important role in de-escalating suicidal crises and facilitating connections to mental health services and community resources [5]. However, much of the existing evidence is observational in nature, and variability in study design and outcome measures limits the ability to draw definitive conclusions regarding long-term effectiveness.

In recent years, attention has increasingly focused on strengthening national behavioral health crisis response systems, including efforts to expand crisis hotline capacity and improve system financing and infrastructure [6]. The implementation of the 988 Suicide & Crisis Lifeline represents one of the most significant structural changes to the U.S. suicide prevention infrastructure in decades. In July 2022, the United States transitioned from the 10-digit National Suicide Prevention Lifeline to the simplified three-digit 988 number, a change intended to improve accessibility, reduce barriers to seeking help, and enhance coordination between crisis hotlines, mobile crisis teams, and crisis stabilization services [7,8].

Early evaluations of the 988 system have reported increases in crisis contacts across calls, texts, and chats since the program’s national launch. National administrative data indicate that millions of contacts have been routed through the 988 Lifeline network, suggesting increased utilization of hotline-based crisis support within the broader behavioral healthcare system [9,10]. At the same time, researchers have emphasized the importance of evaluating system performance at both national and state levels to better understand how local infrastructure, workforce capacity, and public awareness influence crisis system utilization [6].

Public awareness of the 988 Lifeline has increased since its implementation, though knowledge of how the service operates remains more limited. National survey data indicate that while a majority of U.S. adults report having heard of the 988 Lifeline, fewer individuals report understanding when or how to use the service during a mental health crisis [11]. This gap between awareness and functional understanding may contribute to differences in how crisis services are utilized, particularly in communities facing structural barriers to behavioral health care.

Nevada presents a particularly relevant setting for describing operational metrics of the 988 system. The state consistently reports suicide rates higher than the national average, with approximately 690 suicide deaths occurring annually [12]. Rural and frontier communities in Nevada experience a high suicide burden, which has been linked to geographic barriers to care and limited access to behavioral health services [12]. These factors may contribute to increased reliance on crisis intervention services such as 988 for individuals experiencing acute behavioral health crises.

Evaluating the operational performance of the 988 Lifeline within Nevada provides insight into how the national crisis response system functions at the state level and may inform improvements in suicide prevention infrastructure. The objective of this study was to assess operational performance metrics of the 988 Suicide & Crisis Lifeline within Nevada during 2025. Specifically, this study examined monthly call volume, in-state answer rates, call abandonment patterns, average speed to answer, and average talk time to evaluate system utilization and operational performance.

## Materials and methods

Study design and data source

A retrospective descriptive health services analysis was conducted using publicly available operational performance reports from the 988 Suicide & Crisis Lifeline network for calendar year 2025 [10]. These reports provide standardized operational metrics collected from crisis centers participating in the national crisis hotline network and reflect performance indicators defined by the 988 Lifeline system [10].

Monthly state-level reports were accessed through the 988 Suicide & Crisis Lifeline website and downloaded as publicly available PDF documents [10]. Data from January through November 2025 were included in the analysis, as November represented the most recent reporting period available at the time of study completion. While reports from all states were reviewed to provide a national context, the present analysis focused specifically on the Nevada-level operational metrics. National survey data of U.S. adults were reviewed to provide general context regarding public awareness and perceptions of the 988 Lifeline; these data were not directly linked to or analyzed in conjunction with Nevada-specific operational metrics. No additional data sources (e.g., workforce or funding data) were available or incorporated into this analysis.

Variables and data extraction

Nevada-specific operational metrics were extracted from each monthly report. The primary variables examined included call volume (defined as the number of crisis hotline calls routed to Nevada-based crisis centers), in-state answer rate (defined as the proportion of routed calls answered by Nevada crisis centers), and call abandonment rate (defined as the proportion of calls disconnected by the caller before connection with a crisis counselor).

Additional operational performance indicators included average speed to answer (defined as the time between entry into the in-state queue and connection with a counselor) and average talk time (defined as the duration of the interaction once a call had been answered). All measures reflect standardized operational performance metrics as reported by the 988 Lifeline network for calls routed to crisis centers within Nevada.

Data analysis

Data were organized by month and analyzed descriptively to evaluate temporal patterns in system utilization and operational performance. Summary measures, including monthly values, means, ranges, and total call volume, were calculated. Trends in call volume, answer rates, response time, and call duration were visualized using graphical representations across the study period. All calculations and figure generation were performed using Microsoft Excel (Microsoft Corporation, Redmond, WA, USA).

Ethical considerations

This study utilized publicly available, aggregated operational data reported by the 988 Suicide & Crisis Lifeline network. No individual-level or identifiable caller information was accessed. Although the data originate from human crisis hotline interactions, all information analyzed in this study was fully de-identified and reported in aggregate form. Therefore, institutional review board approval was not required.

## Results

In-state answer rate and call abandonment

During the study period (January-November 2025; 11 reporting months), in-state answer rates ranged from 73.19% to 88.67%, with a mean monthly answer rate of 81.2%. The lowest answer rate occurred in February (73.19%), while the highest occurred in August (88.67%). Answer rates increased during the mid-year period and remained elevated through the fall months.

**Figure 1:**
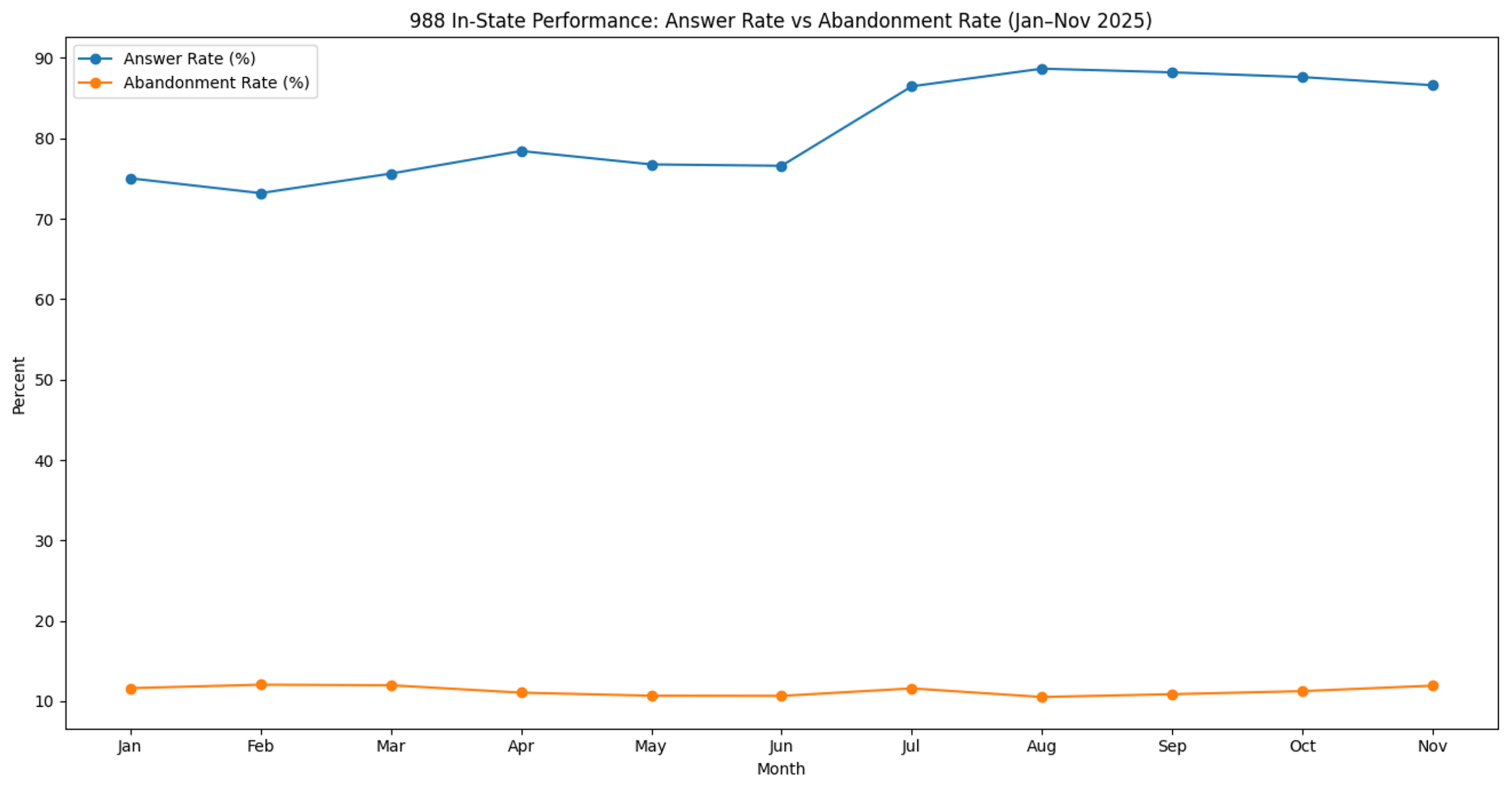
Monthly in-state answer rate and call abandonment rate for the Nevada 988 Suicide & Crisis Lifeline from January through November 2025. Answer rate represents the percentage of routed in-state contacts answered by Nevada crisis centers, while abandonment rate represents the percentage of routed contacts disconnected before being answered. Data were obtained from publicly available monthly in-state key performance indicator reports from the 988 Suicide & Crisis Lifeline network [10]. The figure was generated by the authors using aggregated monthly performance data.

Call abandonment rates remained relatively stable throughout the study period, ranging from 10.53% to 12.06%, with a mean monthly abandonment rate of 11.3%. The lowest abandonment rate occurred in August (10.53%), while the highest occurred in February (12.06%). Monthly trends in in-state answer rates and call abandonment rates are presented in Figure 1.

Call volume

A total of 42,268 calls were routed to Nevada in-state crisis centers through the 988 Suicide & Crisis Lifeline between January and November 2025. Monthly call volume ranged from 3,267 calls in February to 4,295 calls in July, with a mean monthly call volume of approximately 3,843 calls.

**Figure 2:**
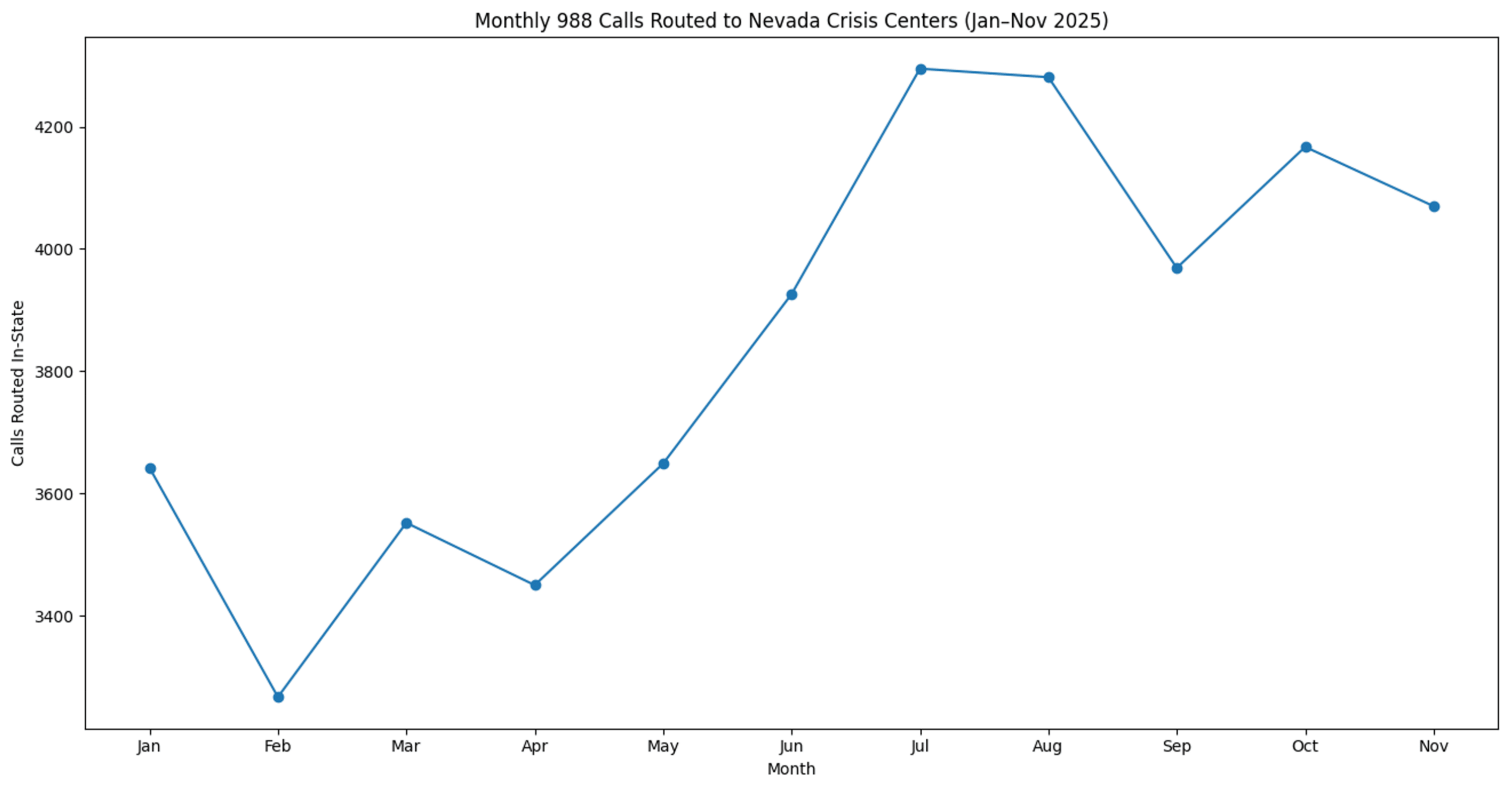
Monthly number of calls routed to Nevada in-state crisis centers through the 988 Suicide & Crisis Lifeline from January through November 2025. Values represent the total number of routed in-state contacts received by Nevada crisis centers during each reporting month. Data were obtained from publicly available monthly in-state key performance indicator reports from the 988 Suicide & Crisis Lifeline network [10]. The figure was generated by the authors using aggregated monthly performance data.

Call volume increased during the mid-year period, peaking in July before stabilizing in the later months of the study period. Monthly variation in routed call volume is presented in Figure 2.

Average speed to answer

Average speed to answer represents the time between when a routed contact enters the in-state queue and when it is answered by a crisis counselor. During the study period, average speed to answer ranged from 17.3 seconds in January to 34.2 seconds in July, with a mean response time of approximately 25.7 seconds.

**Figure 3:**
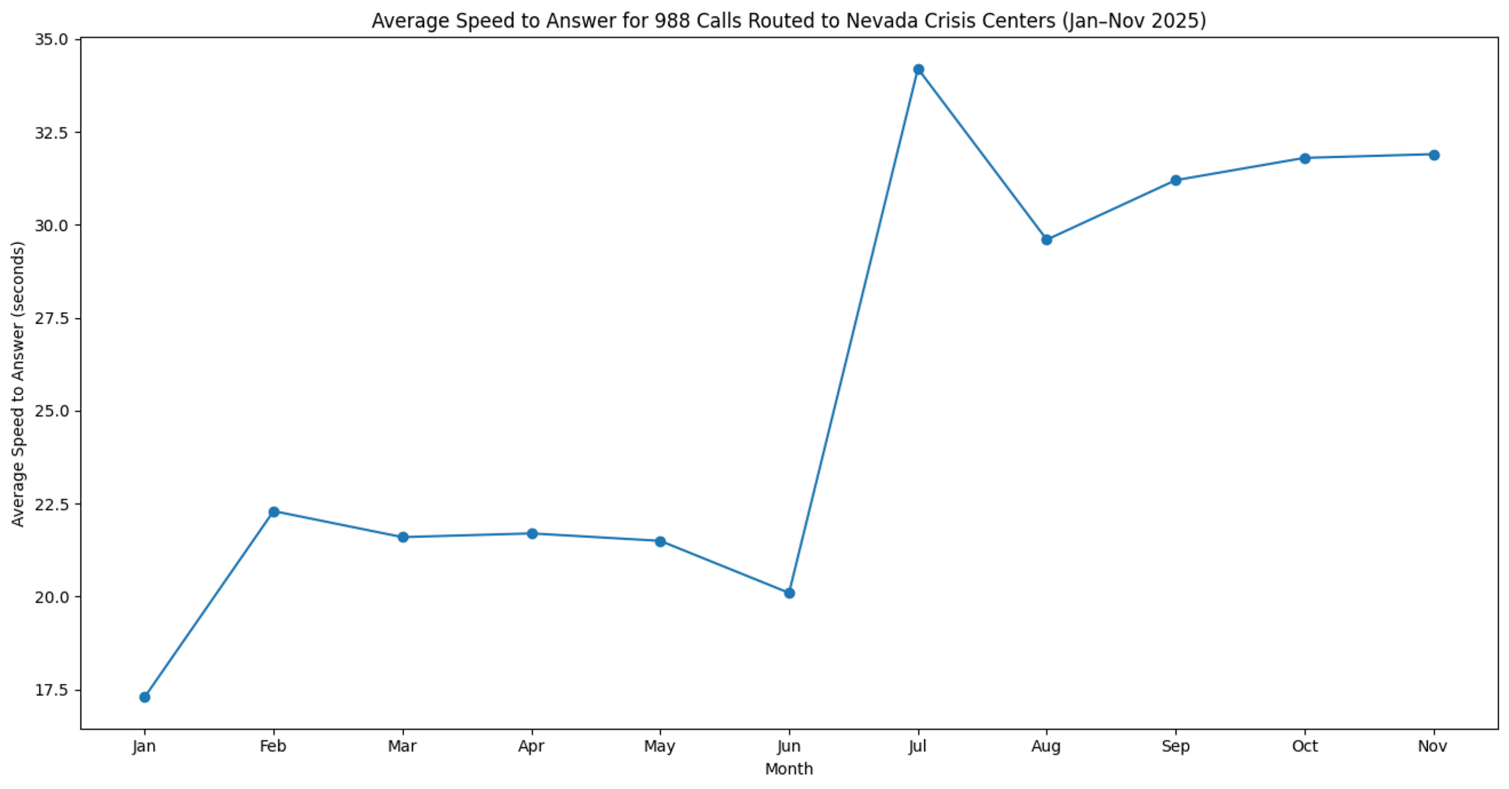
Average speed to answer for calls routed to Nevada crisis centers through the 988 Suicide & Crisis Lifeline from January through November 2025. Values represent the average time in seconds between when a routed contact entered the in-state queue and when it was answered by a crisis counselor. Data were obtained from publicly available monthly in-state key performance indicator reports from the 988 Suicide & Crisis Lifeline network [10]. The figure was generated by the authors using aggregated monthly performance data.

Response times were higher during the mid-year period before declining modestly in the later months, though they remained higher than those observed at the beginning of the year. Monthly trends in average speed to answer are presented in Figure 3.

Average talk time

**Figure 4:**
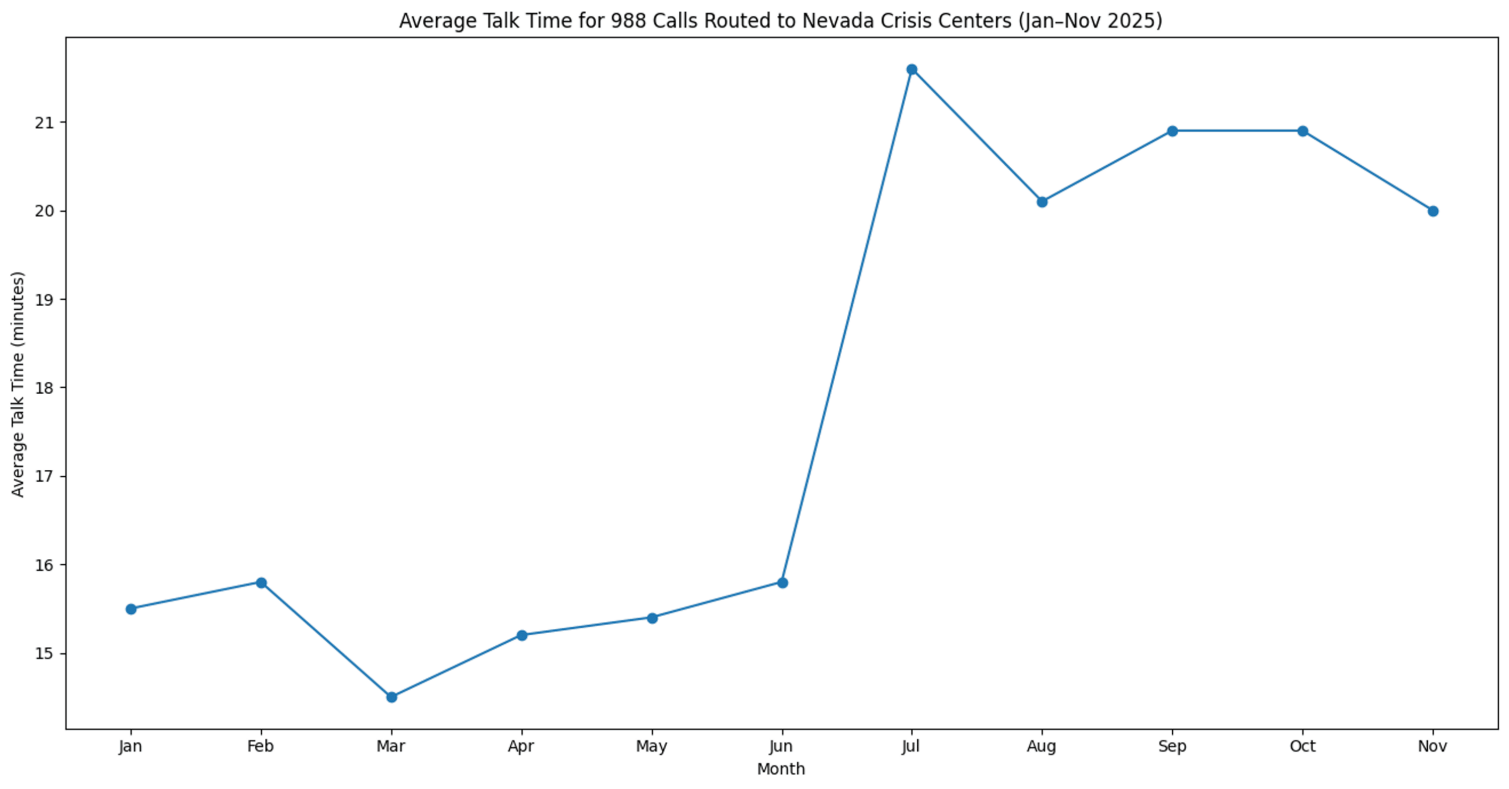
Average talk time for calls routed to Nevada crisis centers through the 988 Suicide & Crisis Lifeline from January through November 2025. Values represent the average duration in minutes between when a routed contact was answered by a crisis counselor and when the call concluded. Data were obtained from publicly available monthly in-state key performance indicator reports from the 988 Suicide & Crisis Lifeline network [10]. The figure was generated by the authors using aggregated monthly performance data.

Average talk time represents the duration of crisis calls once a contact has been answered by a crisis counselor. Average talk time ranged from 14.5 minutes in March to 21.6 minutes in July, with a mean talk time of approximately 17.8 minutes. Following the mid-year increase, talk time remained elevated during the later months of the study period. Monthly trends in average talk time are presented in Figure 4.

## Discussion

Analysis of Nevada’s 2025 988 Lifeline data demonstrates sustained utilization of the crisis hotline throughout the study period, with more than 3,200 calls routed in-state each month. These findings suggest that the 988 Lifeline is functioning as an important component of Nevada’s behavioral health crisis response infrastructure. National evaluations of the 988 system similarly report substantial increases in crisis contacts across calls, texts, and chats following the implementation of the three-digit dialing code, reflecting growing reliance on hotline-based crisis support within the broader behavioral healthcare system [8,9]. Together, these trends support the role of the 988 Lifeline as a key access point within the behavioral health crisis continuum, providing entry into crisis support services for individuals experiencing acute psychological distress. As this analysis is based on aggregated operational data, observed trends cannot be attributed to specific underlying factors such as staffing levels, system capacity, or external environmental influences.

Operational performance metrics further illustrate how the crisis response system functioned during this period. In-state answer rates ranged from approximately 73% to 89% during the study period, while call abandonment rates remained relatively stable between roughly 10% and 12% (Figure 1). Improvements in answer rates observed during the mid-year period coincided with changes in operational conditions within crisis centers over time; however, factors such as staffing levels and system workflows were not directly measured in this analysis. Relatively stable abandonment rates suggest that access to crisis counselors was generally maintained despite fluctuations in call volume.

Monthly variation in call volume was observed, with peak utilization occurring during the summer months of July and August (Figure 2). Seasonal patterns in suicidal behavior and mental health crises have been documented in multiple epidemiological studies, with several analyses demonstrating increased suicide risk during late spring and summer months [13,14]. Environmental and social stressors, including extreme temperatures, seasonal employment fluctuations, disrupted sleep patterns, and reduced social connectedness, have been associated with increased psychological distress during these periods [13,14]. While these findings provide potential context for the seasonal trends observed in Nevada, the present study did not directly evaluate the underlying causes of variation in call volume. Factors such as Nevada’s desert climate and tourism-driven economy may represent state-specific contributors; however, these should be considered hypotheses requiring further investigation rather than established explanations.

Additional operational indicators provide insight into the responsiveness of the crisis response system. Average speed to answer increased during the mid-year period before declining modestly in later months (Figure 3). Although response times varied throughout the year, callers were generally connected to crisis counselors within 17.3 to 34.2 seconds (mean = 25.7 seconds). These response times suggest that crisis centers were largely able to maintain timely access to services despite increased utilization during peak months. Average talk time also increased during the mid-year period and remained elevated thereafter (Figure 4).

Longer call durations may be influenced by factors such as increased complexity of crisis interactions, more comprehensive counselor engagement, evolving crisis center practices, counselor experience, or call characteristics, none of which were directly measured in this study. Evidence from suicide prevention research indicates that crisis interventions, including real-time counseling and chat-based support, are associated with improvements in emotional distress and perceived support among individuals in crisis [5,15]. Structured interventions such as collaborative safety planning have also been shown to reduce suicidal behavior and improve patient safety during acute behavioral health crises [16].

The findings of this study also highlight the relationship between public awareness and functional utilization of crisis services. National survey data indicate that while public awareness of the 988 Lifeline has increased substantially since its implementation, fewer individuals report understanding when or how the service should be used during a behavioral health crisis [11]. This gap between awareness and functional understanding may limit optimal utilization of crisis services, particularly among individuals experiencing emerging psychological distress who may not yet perceive their situation as warranting crisis intervention. Improving public education regarding the role of 988 as a confidential resource for emotional support, suicide prevention, and behavioral health crises may therefore enhance earlier engagement with crisis care systems.

Existing psychiatric research supports the effectiveness of crisis hotline interventions in reducing acute distress and suicidal ideation among callers. Observational studies of suicide prevention hotline interactions have consistently demonstrated reductions in emotional distress and suicidal urgency during crisis calls [3,4]. In addition, systematic reviews evaluating crisis hotline services report that many callers perceive the interaction as helpful and experience increased feelings of hope following engagement with trained counselors [5]. More recent analyses of crisis chat services similarly indicate improvements in emotional distress and perceived support following interaction with trained counselors [15]. Structured crisis interventions such as collaborative safety planning have also been shown to reduce suicidal behavior and improve patient safety during acute behavioral health crises [16].

In states such as Nevada, where geographic barriers may limit access to behavioral health care, crisis hotline services may serve as an important entry point into the mental health system. Nevada consistently reports elevated suicide rates relative to the national average [12]. In such settings, crisis hotlines can provide immediate access to trained counselors while facilitating connections to local mental health resources, including mobile crisis teams and community-based behavioral health services.

Policy implications

The findings of this analysis suggest several opportunities for strengthening behavioral health crisis response systems at both the state and national levels. Continued investment in crisis center workforce capacity may be important for maintaining high in-state answer rates as public awareness of the 988 Lifeline continues to increase. Ensuring adequate staffing and training for crisis counselors may be particularly important during periods of increased demand. Expanding public education campaigns that clarify when and how individuals should use the 988 Lifeline may improve earlier engagement with crisis services and reduce barriers to seeking support.

In addition, strengthening coordination between crisis hotlines, mobile crisis response teams, and crisis stabilization services may further improve the effectiveness of behavioral health crisis systems by facilitating timely connection to in-person care when needed. National policy frameworks, including the National Guidelines for Behavioral Health Crisis Care and the Crisis Now model, emphasize the importance of integrating crisis hotlines, mobile crisis teams, and stabilization services into a comprehensive behavioral health crisis care continuum capable of providing timely, community-based responses to individuals experiencing acute psychological distress [17-19].

Limitations

Several limitations should be considered when interpreting these findings. First, this study relied on aggregated operational data from publicly available 988 Lifeline performance reports and therefore did not include individual-level caller information or demographic characteristics. As a result, it was not possible to evaluate how utilization patterns varied by age, gender, geographic location, clinical characteristics, or other contextual factors influencing crisis service use, including staffing levels, workforce training, system capacity, or funding changes over time. Additionally, this study did not include counselor-level data, such as provider experiences, perceptions, or the potential psychological impact of crisis intervention work.

Second, the analysis was descriptive in nature and did not include statistical testing or allow for causal or inferential conclusions regarding factors influencing crisis hotline utilization or operational performance. Third, the study period included data from January through November 2025, as December reports were not yet available at the time the analysis was conducted. Finally, while national survey data were used to contextualize findings related to awareness and perceived effectiveness of the 988 system, these data were not directly linked to Nevada-specific hotline utilization patterns.

## Conclusions

In 2025, the 988 Suicide & Crisis Lifeline demonstrated sustained utilization in Nevada, with consistent call volumes and stable in-state answer rates observed across the study period. Monthly variation in call volume, including increased utilization during summer months, highlights potential seasonal demand patterns that may inform future capacity planning. While national awareness of 988 is high, gaps in public understanding of how and when to use the service remain. Continued investment in crisis center workforce capacity, targeted public education, and ongoing evaluation of system performance may support further development of behavioral health crisis response systems in Nevada.
